# How Robust is the Evidence for a Role of Oxidative Stress in Autism Spectrum Disorders and Intellectual Disabilities?

**DOI:** 10.1007/s10803-020-04611-3

**Published:** 2020-09-14

**Authors:** Shanna L. Burke, Jessica Cobb, Rumi Agarwal, Marlaina Maddux, Marcus S. Cooke

**Affiliations:** 1 BRAINN Lab, School of Social Work, Robert Stempel College of Public Health and Social Work, Florida International University, 11200 S.W. 8th Street, Miami, FL 33199 USA; 2 Oxidative Stress Group, Department of Environmental Health Sciences, Florida International University, 11200 S.W. 8th Street, Miami, FL 33199 USA; 3 FIU Embrace, Florida International University, 11200 S.W. 8th Street, Miami, FL 33199 USA; 4 Department of Health Promotion and Disease Prevention, Robert Stempel College of Public Health and Social Work, Florida International University, 11200 S.W. 8th Street, Miami, FL 33199 USA; 5 Biomolecular Sciences Institute, Florida International University, 11200 S.W. 8th Street, Miami, FL 33199 USA; 6 Present Address: Easterseals Blake Foundation, Tucson, AZ USA

**Keywords:** Oxidative stress, Intellectual disability, Developmental disability, Autism spectrum disorder, Biomarker

## Abstract

Growing interest in the pathogenesis of autism spectrum disorders (ASDs) and other intellectual and developmental disabilities (IDD) has led to emerging evidence implicating a role for oxidative stress. However, understanding the strength of this association is made challenging by the use of a variety of purported biomarkers of oxidative stress, many of which have either uncertain specificity or flawed methods of analysis. This review aims to address this issue, which is widespread in the ASD and IDD literature, by providing readers with information concerning the strengths and limitations of the choice and analysis of biomarkers of oxidative stress. We highlight that biomarkers and assays should be specific, sensitive, reproducible, precise, robust, and chosen with careful consideration. Future studies should be sufficiently powered and address sample collection, processing, and storage which are, additionally, poorly considered, sources of bad practice, and potential errors. Only with these issues considered, will the data lead to conclusions as to the precise role of oxidative stress in ASDs and IDD.

Autism spectrum disorders (ASDs) and intellectual and developmental disabilities (IDD) are both neurodevelopmental disorders, which include but are not limited to Down syndrome and Fragile X syndrome, with varying levels of severity. ASDs affect one in 54 children in the United States and are 4.3 times more likely to develop in boys versus girls (Maenner et al. 2020). While it is unknown precisely how many individuals are affected by IDD, the American Academy of Pediatrics (2017) estimates that nearly 6.5 million individuals in the United States have some level of IDD diagnosis. Although the biological basis for ASDs and IDD is as yet unknown, recently, due to an interest in determining the pathogenesis of these disorders, there has been a focus on pediatric populations with neurodevelopmental disorders. This has led to an emerging body of evidence implicating that oxidative stress (arising from an imbalance between prooxidants and antioxidants, in favor of the former), and the resulting damage to cellular molecules, has a mechanistic role in ASDs and IDD (Adams et al. 2009; Belardinelli et al. 2008; Chauhan et al. 2004; Cooke et al. 2003; De Felice et al. 2012; Gebril and Meguid 2011; Ghezzo et al. 2013; Grillo et al. 2013; James et al. 2004, 2006; Rose et al. 2012; Sajdel-Sulkowska et al. 2011; Vergani et al. 2011). This is plausible, as the brain has a large oxidizing capacity with multiple sources of reactive oxygen species (ROS), and is reported to not have a matched ability to combat oxidative stress, creating a vulnerability to neurodegeneration (Ansari and Scheff 2010; Martínez et al. 2013). As a result, oxidative stress-derived damage to cellular biomolecules (nucleic acids, lipids, and proteins, specifically) can result in neuronal dysfunction and brain tissue loss (Forster et al. 1996; Harman 1956; Liu et al. 2017; Shan and Lin 2006). Despite the recent attention to the connection between oxidative stress, ASDs, and IDD in the pediatric population (Frustaci et al. 2012), a comprehensive assessment in a well-phenotyped adult cohort of individuals remains sparse.

Oxidative stress arises from the excessive production of reactive oxygen species (ROS), which originates from several exogenous and endogenous sources, such as normal metabolism, inflammation, or the reduction of antioxidant defenses, or a combination thereof (Biswas 2016; Cooke et al. 2003). While ROS production can be challenging to quantify directly, particularly in vivo, oxidative stress can be evaluated by measurement of the damage to cellular biomolecules (e.g. nucleic acids, lipids, and proteins). The measurement of such biomarkers has been widely reported, establishing links between oxidative stress and a variety of diseases (Evans et al. 2004). For example, 8-oxo-7,8-dihydroguanine (8-oxoGua; and its corresponding 2′-deoxyribonucleoside, 8-oxo-7,8-dihydro-2′-deoxyguanosine, 8-oxodG) is a well-established, and widely measured biomarker of oxidative stress (Lam et al. 2012). 8-oxoGua can be measured in the DNA of solid tissues or, less invasively, in white blood cells. However, urine represents a non-invasive matrix in which to assess oxidative stress and, as such, decreases the concerns of ethical review boards, particularly when considering studying vulnerable populations.

This review aims to provide a critical evaluation of the use of biomarkers of oxidative stress in ASD and IDD research. In particular, we have highlighted representative studies that illustrate the use of less than robust biomarkers or assays. By considering biomarker measurement in blood, urine, and tissue, and critically assessing the choice of biomarker, and method of analysis, we have extended this work well beyond the systematic review of blood-based biomarkers of Frustaci et al. (2012) and Kałużna-Czaplińska and Jóźwik-Pruska's (2016) review of (chromatographic and mass spectrometric) methods of studying oxidative stress in ASD, including making recommendations for the more robust biomarkers, and most appropriate biomatrices, for the assessment of oxidative stress (Table 1).

**Table 1:** General guide for the selection of biomarkers of oxidative stress in in vivo ASD studies

Biomarker	Biological matrix	Method of analysis	Notes
*Nucleic acids* e.g. 8-oxodG, 8-oxoGuo	*Urine* Requires correction for variation in urine concentration/output e.g. 24 h collections, or correction for creatinine, or specific gravity	ELISA	The literature reports both in-house, and commercially available ELISAs, typically for the (semi-)quantification of 8-oxodG *Advantages* assay is simple to use, little sample work-up, high throughput, kits, and associated equipment cheaper than HPLC–MS/MS *Disadvantages* lack of absolute quantification, and internal standardisation; generally, the ELISA approach over-estimates, compared to HPLC–MS/MS; potential lack of specificity; limited to one analyte per kit
		HPLC–MS/MS	Considered to be the gold standard approach *Advantages* absolute quantification, use of stable isotopically-labelled internal standards account for target loss during sample workup, and matrix effects; multiple (> 1) analytes can be analysed simultaneously e.g. 8-oxodG and 8-oxoGuo *Disadvantages* cost—initial cost of equipment, service contracts, requires specialist training for operator; may occupy considerable laboratory space
		HPLC-ECD	Rarely reported in the literature, as it has largely been replaced by LC–MS/MS *Advantages* more specific than ELISA, *Disadvantages* lacks internal standardization—less accurate than HPLC–MS/MS; analytes must be electrochemically active e.g. 8-oxodG
	*Blood: whole blood, serum, or plasma* if aiming to study a nucleic acid-derived biomarker of oxidative stress in serum or plasma of these matrices, it is worth considering using urine instead, as it possesses the advantage of being entirely non-invasive	ELISA	*Advantages* as above, for urine *Disadvantages* as above, for urine
		HPLC–MS/MS	*Advantages* as above, for urine *Disadvantages* as above, for urine
		Comet assay—whole blood	*Advantages* as little as 5 µL of whole blood contains sufficient cells for use in the comet assay; this blood can be added to a gel with no workup, no isolation of peripheral blood mononuclear cells; minimally invasive (a pin-prick of blood from a lancet provides sufficient blood). Can detect double-, and single-strand breaks (at neutral pH), plus alkali-labile sites at alkali pH. Use of DNA repair enzymes can provide specificity for certain nucleobase lesions, although their precise substrate specificity may not be known) *Disadvantages* semi-quantitative, unless calibration is used; precise nature of lesions undefined
	*Solid issue* generally, obtaining tissue is necessarily invasive	ELISA	*Advantages* as above, for urine *Disadvantages* requires DNA extraction with attendant risk of artefact; extracted DNA may be applied to the ELISA, but quantification not possible as the polymeric DNA is not in the same structural form as the external calibration curve (the free modified 2′-deoxyribonucleoside, 8-oxodG)
		HPLC–MS/MS	*Advantages* as above, for urine *Disadvantages* requires DNA extraction with attendant risk of artefact; DNA requires hydrolysis to 2′-deoxyribonucleosides—risk on incomplete hydrolysis, and artefact formation;
		HPLC-ECD	*Advantages* as above, for urine *Disadvantages* requires DNA extraction with attendant risk of artefact; DNA requires hydrolysis to 2′-deoxyribonucleosides—risk on incomplete hydrolysis, and artefact formation;
		Comet assay	*Advantages* does not require DNA extraction, and hydrolysis *Disadvantages* requires a single cell suspension to be generated from the tissue
		Immunohisto/cytochemistry	*Advantages* provides localisation of damage within tissues/cells *Disadvantages* at best semi-quantitative; protocols can be challenging e.g. immunohistochemistry for 8-oxodG
*Lipids* e.g. malondialdehyde (MDA), 4-hydroxy-2-nonenal (HNE), F2-isoprostanes (F2-IsoPs)	*Urine* requires correction for variation in urine concentration/output e.g. 24 h collections, or correction for creatinine, or specific gravity	ELISA	*Advantages* assay is simple to use, little sample work-up, high throughput, kits, and associated equipment cheaper than HPLC–MS/MS *Disadvantages* antibody-based methods do not compare well with mass spectrometric methods
		HPLC–MS/MS	HPLC–MS/MS is considered the gold standard method for the analysis of isoprostanes, specifically 8-iso-IPF2α *Advantages* absolute quantification, use of stable isotopically-labelled internal standards account for target loss during sample workup, and matrix effects; multiple (> 1) analytes can be analysed simultaneously *Disadvantages* cost—initial cost of equipment, service contracts, requires specialist training for operator; may occupy considerable laboratory space. Artefactual formation of isoprostanes occurs during sample storage at − 20 °C, but can be prevented for up to six months by storage at − 80 °C, or addition of a preservative, such as butylated hydroxytoluene
		HPLC–UV	HNE can be quantified either directly, of following derivatisation with 2,4-dinitrophenylhydrazine (DNPH) *Advantages* a relatively simple assay *Disadvantages* lacks sensitivity
		HPLC-fluorescence	The thiobarbituric acid reactive substances (TBARS) assay is often described as detecting MDA (although alkenals and alkadienals are also TBARS), however, HPLC with fluorescence detection is required in order to identify and quantify the MDA-TBA adduct, and discriminate from all the TBA-reactive substances (e.g. sugars, amino acids, bilirubin, and albumin) that are otherwise detected colorimetrically *Advantages* provides specificity for MDA-TBA adduct impossible with the simple colorimetric assay (which is often marketed commercially as a test for lipid peroxidation or MDA) *Disadvantages* lack of internal standardisation; calibration curve with authentic MDA standard cannot be generated due to instability of MDA; dietary intake of fatty acids influences MDA levels; MDA is a by-product of prostaglandin synthesis
	*Blood* whole blood, serum, or plasma	HPLC-fluorescence	See above for MDA measurement in urine *Advantages* provides specificity for MDA-TBA adduct impossible with the simple colorimetric assay (which is often marketed commercially as a test for lipid peroxidation or MDA) *Disadvantages* invasive; also see urine
		HPLC–MS/MS	Half-life of isoprostanes in plasma is ~ 18 min, this is circumvented by measuring 2,3-dinor 8-iso-PGF1a, the metabolite of iso-PFG2α *Advantages* see urine *Disadvantages* see urine
		GC–MS	HNE can be quantified following derivatisation with 1,3-cyclohexanedione *Advantages* specific, sensitive, absolute quantification. Potential for use of stable isotope internal standards, to improve quantification, and account for matrix effects *Disadvantages* F2-IsoPs and MDA results can be affected by sample haemolysis
	*Solid tissue*	Schiff’s reaction	*Advantages* localisation of aldehydes within a tissue *Disadvantages* questionable specificity for lipid peroxidation-derived aldehydes
		GC–MS	*Advantages* sensitive and specific for isoprostanes *Disadvantages* isoprostanes require extraction from tissue, and then derivatisation, adding to run time
*Proteins* e.g. protein carbonyls, bityrosine, L-DOPA, *ortho*-tyrosine	*Urine* requires correction for variation in urine concentration/output e.g. 24 h collections, or correction for creatinine, or specific gravity	ELISA	Several antibodies exist for the protein carbonyl-DNPH conjugate, and can be applied semi-quantitatively to ELISA, or Western blotting (after 1 or 2D electrophoresis) *Advantages* assay is simple to use, little sample work-up, high throughput, kits, and associated equipment cheaper than HPLC–MS/MS. Sensitive, small sample volume, reproducible, and large sample throughput *Disadvantages* relies upon specificity of the primary antibody; semi-quantitative
		HPLC–MS/MS	e.g. bityrosine Advantages: sensitive and specific quantification *Disadvantages* see above for lipids. May require derivatization with DNPH followed by solid phase extraction
		HPLC with fluorescence detection (protein carbonyls), or chemiluminescence (protein hydroperoxides)	*Advantages* provides specificity for protein carbonyl-dinitrophenyl hydrazine conjugate, which is impossible with the simple colorimetric assay *Disadvantages* risk of protein oxidation during sample work-up; lack of internal standardisation
	*Blood* plasma/serum	ELISA	See above for urine
		HPLC–MS/MS	See above for urine
		HPLC with fluorescence detection (protein carbonyls), or chemiluminescence (protein hydroperoxides)	See above for urine
		Colorimetric assay	Requires sample to be derivatized with DNPH prior to spectrophotometric measurement *Advantages* low cost *Disadvantages* does not provide any information on which protein has been oxidised
	*Solid tissue* Generally, obtaining tissue is necessarily invasive	Histochemistry	Blood (plasma/serum), and urine are the preferred matrices
			Protein carbonyls are derivatised with 2,4-DNP, the resulting 2,4-dinitrophenyl hydrazine then detected by a commercial anti-DNP antiserum *Advantages* localisation of protein oxidation within a tissue *Disadvantages* does not provide any information on which protein has been oxidised

For biomarkers of oxidative stress, additional logistical considerations may include: method, and regularity, of sampling; sample preservation, pre-processing, and storage (type, temperature, and duration of storage); status of biomarker, and assay, validation; biomarker variability (inter- and intra-individual, and assay variability). When biomarkers are measured in a surrogate matrix for the target tissue, consideration must be given as to what these measurements reflect

## Methods

The purpose of this study was to critically review the literature relating to the use of biomarkers of oxidative stress in ASD and IDD, and highlight the attention of the reader to best practices concerning the analysis of these biomarkers. Rather than a systematic review procedure, this review sought to obtain and synthesize representative, relevant articles on the topic, though this was limited by availability, language (English language was necessary for review), and relevance to the topic when the article was reviewed in full. The inclusion criteria for this review stipulated that a study must discuss oxidative stress amongst individuals (children or adults) with ASD or IDD and be published between the years 1997 and 2018. Studies involving animal models were included only for studies amongst IDD, given the sparse state of the evidence.

Published peer-reviewed manuscripts were identified using electronic databases available through a large University library. With subscriptions to over 1147 databases, the electronic databases of EMBASE, Medline and PsycINFO were the primary means of obtaining articles, though sources such as google scholar, searching of references in cited manuscripts, and alternative means of obtaining articles (interlibrary loan requests, ResearchGate, and contacting authors directly) were used if the full-text was not available for review. Search strategies were customized for each database using controlled vocabulary related to “oxidative stress,” “autism spectrum disorder,” and “intellectual and developmental disability.”

## Results

The findings from the review have been presented below concerning the two diagnoses of interest—ASD and more generally, IDD. A careful review of the resulting literature revealed a concerningly large number of studies with methodological limitations (discussed as part of this review), together with some studies which mentioned oxidative stress in the title/abstract, but did not actually perform any such analyses e.g. Geier et al. (2019), or was fundamentally flawed due to an “n” of one, or demonstrated a use of obscure methodology e.g. d-ROMS (Reactive Oxygen Metabolites) Test (Messina et al. 2018). In the latter study, the authors incorrectly described oxidized nucleic acids as ROS and used an undescribed, unreferenced photometric assay to measure plasma hydroperoxides in serum or plasma (the authors used both terms, seemingly interchangeably). Curiously, rectal temperature was one of the outcomes measured (the significance of which was not reported). Other studies also omitted sufficient information concerning the methodology used to make an accurate assessment e.g. El-Ansary et al. (2017).

### Oxidative Stress and ASD

Children with ASD have been found to have elevated levels of lipid peroxidation, a marker of increased oxidative stress in plasma, compared to their neurotypical siblings. Plasma (malondialdehyde, MDA; an end product of lipid peroxidation) was found to be increased in 87% of the children with ASD. Furthermore, levels of transferrin and ceruloplasmin in plasma were highly correlated with a loss of previous language skills in this group, suggesting an important role for abnormal iron and copper metabolism in ASDs (Chauhan et al. 2004). As such, researchers have suggested the presence of abnormal iron and copper metabolism in ASDs may be responsible for the increased oxidative stress, and therefore have a pathogenic role in ASDs (Arora et al. 2017; Chauhan et al. 2004, 2008; Grabrucker et al. 2016; Sayehmiri et al. 2015), although consensus has not been reached in this area (Fluegge 2017).

Similarly, in children under the age of 6, superoxide dismutase (SOD) and glutathione peroxidase was reduced, while MDA was increased, although these trends did not persist in children over the age of 6 in either group (Gebril and Meguid 2011). A recent study by Yenkoyan et al. (2018) however, used several markers to measure chronic inflammation amongst 10 children (4–10 years) diagnosed with ASD and their siblings. Diminished SOD and enhanced catalase (CAT), which led to a significant decrease in the SOD/CAT ratio and increased carbonyl content in the plasma of patients with ASD, which was not noted in the control group. Furthermore, a negative correlation was seen between SOD activity, urinary 8-oxodG, and respiratory burst intensity in peripheral blood polymorphonuclearleucocytes in ASD participants. However, other studies have shown positive associations between serum and urinary 8-oxodG and ASD severity (Osredkar et al. 2019), and diagnosis (Metwally et al. 2018), respectively.

In addition, low levels of SOD in children with ASDs have been positively correlated with the magnesium content in red blood cells, which potentially implicates mitochondrial MnSOD and the role of mitochondrial dysfunction in the etiology of ASD. The study concluded that the “SOD/CAT imbalance may play a role in the pathogenesis of ASD, leading to enhanced ROS leakage, oxidatively generated damage to macromolecules, chronic/persistent inflammation, and, finally, abnormal neuronal cell functioning or, potentially, to neuronal death” (Yenkoyan et al. 2018).

Ghezzo et al. (2013) evaluated multiple measures of oxidative stress as well as related aspects such as the functional features of the erythrocyte membrane and lipid composition. Erythrocyte thiobarbituric acid-reactive substances (TBARS), urinary isoprostanes, and hexanoyl-lysine adduct levels were all elevated in 21 children (5–12 years) diagnosed with ASD, suggesting an imbalance of the redox status of these children. A marked 66% reduction of Na+ /K+ -ATPase activity was also noted, which, they suggested, could be used as a biomarker of ASD, given that an ASD diagnosis is still based on clinical features, and that no validated physiological biomarkers exist yet for screening or diagnostic purposes (Ghezzo et al. 2013). A more recent study reported that a panel of biomarkers, including lipid peroxides, vitamins C and E, GSH/GSSG, potentially had predictive value in ASD (El-Ansary et al. 2017). However, the use of spectrometric or ELISA methods for these endpoints is likely to weaken the strength of this finding – particularly as extracellular, rather than intracellular, levels of the biomarkers were studied. As we noted for other studies, the reporting of additional methodological details were needed in this study (El-Ansary et al. 2017).

Results from a systematic review and meta-analyses examining blood-based biomarkers related to oxidative stress in ASD found altered pathways for GSH, transmethylation, and transsulfuration. While blood-oxidized glutathione increased, blood GSH, glutathione peroxidase, methionine, and cysteine were reduced (Frustaci et al. 2012). However, many reviews on this subject fail to consider the choice of biomarkers, and whether their method of analysis (plus sample collection, storage, etc.), were appropriate, for example, Rossignol and Frye (2014), and Waligóra et al. (2019). Some studies place considerable emphasis on GSH, as a purported biomarker of oxidative stress, and therefore the glutathione S-transferases (GSTs, as important factors in the antioxidant defense system (Mandic-Maravic et al. 2019), (and references contained in Frustaci et al. (2012)). However, GSH can hardly be described as a specific biomarker of oxidative stress, not least because levels are influenced by the activity of GSTs, whose primary role is in phase II xenobiotic metabolism. Therefore, exposure to endogenous metabolites and xenobiotics will influence GSH levels, independently of oxidative stress.

While cellular 8-oxodG is generally understood to be a biomarker of oxidatively damaged DNA, 3-nitrotyrosine (3-NT) is a biomarker of oxidation generated protein damage (Rose et al. 2012), and both are reported to be significantly increased in the brains (cerebellar hemispheres and putamen) of individuals with ASD (Sajdel-Sulkowska et al. 2011). Increased levels of 3-NT were associated with increased levels of neurotrophin-3 (NT-3) in the cerebella of individuals with ASD, with increased levels in additional brain regions varying between the two brains with autism (Sajdel-Sulkowska et al. 2011).

Some studies examined the effects of oxidative stress in specific brain regions, including the cerebellum and temporal cortex or Brodmann area 22 (BA22). Rose et al., (2012) measured GSH/GSSG (reduced glutathione/oxidized glutathione), 3-NT, 3-chlorotyrosine (3-CT; a marker of inflammation), and 8-oxodG to evaluate whether low GSH redox capacity is associated with increased levels of biomarkers of oxidatively generated damage to protein (i.e. 3-NT), oxidatively damaged DNA (i.e. 8-oxodG), inflammation, and mitochondrial superoxide formation in the cerebellum and BA22 of postmortem brains of individuals with ASD. The results of this study indicate a role of a low GSH:GSSG, and thus oxidative stress, in determining the neuropathology of autism. Furthermore, the results provide evidence that this redox imbalance, in certain brain regions, can lead to further oxidative stress and cell damage via the neuroinflammatory process (Rose et al. 2012). A similar study in a mouse model of ASD (BTBR mice) revealed that deficiencies in antioxidant defense can lead to increased autism-like repetitive behaviors in the mice (Nadeem et al. 2019), although the study was not without its methodology limitations, such as the use of the TBARS assay to assess lipid peroxidation in plasma a cerebella tissue (see “Discussion” for the limitations of this assay). Analysis of 3-NT and 3-CT, together with mitochondrial defects, were used to better classify subgroups of children with ASD, and conclude that their treatment may need to differ based upon their differing metabolic profiles (Frye et al. 2013).

Early research into oxidative stress, toxic metals, and metallothioneins (MT) in individuals with ASDs indicates a relationship between oxidative stress and susceptibility to the effects of certain environmental stressors in the onset and severity of ASD. Vergani et al. (2011) observed significantly higher levels of five essential elements (calcium, zinc, silicon, nickel, and iron) and two toxic metals (cadmium and arsenic) in the plasma of children with ASDs. The same study reported elevated levels of MT mRNA in the plasma of children with ASDs (Vergani et al. 2011). Oxidative stress is a potential trigger for increased MT expression, which increases susceptibility to toxic metals. While research on the role of environmental toxic metals on oxidative stress in ASD is still relatively limited, a recent study sought to further elucidate the relationship; Arora et al. (2017) used the baby teeth of 32 pairs of twins and 12 individual twins to measure if pre- and postnatal exposure to metals such as manganese, lead and zinc increase ASD risk. The population sample included twins where one, both, or neither had ASD to control for genetic factors given that 50% of ASD risk is estimated to be attributed to genetics, and 50% of ASD risk may be due to environmental factors or gene/environment interactions. Using laser-ablation-inductively coupled plasma mass spectrometry the results demonstrated differences in metal uptake between individuals with ASD and siblings without ASD, but only during specific developmental periods. Individuals with ASD had elevated levels of lead when compared to their twin 10 to 20 weeks after birth, although the greatest differences between the infants with ASD and their siblings was at 15 weeks after birth. The infants with ASD showed lead levels 1.5 times higher than their twins. Manganese and zinc levels were both lower in infants with ASD. Manganese was lower (statistically significant) over two important time periods: 1) 10 weeks prior to birth, and 2) 5–20 weeks after birth. The greatest difference was noted 15 weeks after birth when infants with ASD had 2.5 times lower manganese than their twin. Zinc levels were also insufficinet in individuals with ASD from 10 weeks prior to birth to approximately 5 weeks after birth (Arora et al. 2017). Given that clinical signs of ASD begin to present 6–12 months after birth, this study highlights the likelihood of multiple mechanisms, such as environmental exposures and genetics, which can contribute to dysregulation. These findings suggest that the mid-to-late-fetal stage is a crucial time point in the pathophysiological mechanisms of ASD etiology. These results are consistent with the premise that oxidative stress increases susceptibility to environmental exposure, such as toxic metals, or (perhaps more likely) vice versa, that environmental exposures, such as toxic metals, act via oxidative stress.

Other environmental factors such as maternal immune conditions like maternal diabetes, a high-fat diet, and obesity have also been examined as a result of changes brought about in the intrauterine environment. In a review paper (Carpita et al. 2018), the association of these conditions on oxidative stress and ASD has been mixed. However, one specific systematic review and meta-analysis on ASD concluded “that maternal diabetes acts as a risk factor (OR 1.48) without significant heterogeneity (*I*^2^ = 9.1, *p* = 0.35)” (Xu et al. 2014).

### Oxidative Stress and IDD

The literature describing the association between oxidative stress and IDD is more limited than that for ASD. Studies, though sparse, have begun to describe the potential role of oxidative stress and mitochondrial dysfunction in the pathogenesis of a wide variety of neurodevelopmental conditions, including Fragile X (Lima-Cabello et al. 2016), Rett syndrome (De Felice et al. 2012; Gold et al. 2014; Pecorelli et al. 2013), Down syndrome (DS) (Belardinelli et al. 2008; Evans et al. 2016; Jovanovic et al. 1998; Seidl et al. 1997; Žitňanová et al. 2004), and intellectual disability in general (Carmeli et al. 2012).

#### Fragile X Syndrome

Research to date on the relationship between oxidative stress and Fragile X syndrome is limited; however, one study using gene knockout mice models has been performed utilizing FMR1^−/−^ (Fragile X mental retardation) knock out mice to examine biochemical differences in mice with the syndrome versus controls (el Bekay et al. 2007). Multiple biomarkers of oxidation were measured concurrently in the brain tissue of FMR1^−/−^ and WT control mice, which included 1) free radical production in brain tissue and macrophage cells, 2) nicotinamide adenine dinucleotide phosphate (NADPH)-oxidase activity in total and regional brain samples, 3) TBARS levels, which may relate to lipid damage, and 4) the carbonyl content of proteins. The researchers found increased levels of ROS, TBARS, and carbonyl groups in the brains from FMR1^−/−^ mice (compared to WT). FMR1^−/−^ mice exhibited enhanced NADPH-oxidase activity, antioxidant glutathione levels, and glutathione enzyme activities; processes that may cause deleterious effects in brain tissue. As such, oxidative stress may underlie some of the pathophysiological hallmarks of Fragile X syndrome (el Bekay et al. 2007). An interesting development in the ability to assess protein carbonyls and apply to ASD is the use of redox proteomics to compare multiple plasma protein carbonyls. In a recent study, significantly increased levels of two protein carbonyls (compared to controls) were noted in the plasma of individuals with ASD (Feng et al. 2017).

#### Rett Syndrome

Studies examining biomarkers of oxidative stress in individuals with Rett syndrome found differences between siblings with classical Rett syndrome and the Zappella variant, even though both share the *MECP2 *(OMIM*300005) mutation. HPLC coupled with gas chromatography/negative ion chemical ionization tandem mass spectrometry (GC/NICI-MS/MS) was used to measure non-protein bound iron (NPBI), and isoprostanes respectively (Ciccoli et al. 2003). The results indicated that for NPBI, F(2)-dihomo-isoprostanes (F2-dihomo-IsoPs), F(3)-isoprostanes (F3-IsoPs), F(4)-neuroprostanes (F4-NeuroPs), and F(2)-IsoPs there was statistically significant increased levels in individuals with classical Rett syndrome versus controls, although this was not found in individuals with the Zappella variant (De Felice et al. 2012). Pecorelli et al. (2013) evaluated levels of 4-hydroxynonenal plasma protein adducts (4HNE-PAs) in individuals with Rett syndrome variants. Elevated 4HNE-PAs were reported in *MECP2* and *CDKL5* mutant variants, but not in *FOXG1*, which, the authors claim, indicates the presence of oxidative stress in these variants and supports the hypothesis that it plays a role in its pathogenesis (Pecorelli et al. 2013).

Consistent with findings on oxidative stress and ASDs, there is also evidence for a link between mitochondrial dysfunction in mice with Rett syndrome. When examining a mouse model with symptomatic Rett syndrome, the mitochondrial respiratory chain enzyme activity of complexes II, III, IV, and GSH levels were significantly decreased in mice with Rett syndrome characteristics, compared to the pre-symptomatic mice. The hypothesis that decreased GSH levels leads to an accumulation of free radicals, and results in oxidative stress in mice with Rett syndrome is consistent with the hypothesis that mitochondrial dysfunction is linked to the pathogenesis of this disease. This further suggests that medical management of cellular GSH levels, e.g. via supplementation, may mitigate the effects of oxidative stress on neurological and motor deterioration in individuals with Rett syndrome (Gold et al. 2014).

#### Down Syndrome

Down syndrome, which arises from a third copy of chromosome 21 and carries with it deficits in intellectual ability, has been correlated with increased oxidative stress (determined by urinary levels of 8-oxodG; Jovanovic et al. 1998) although a separate study examining 8-oxodG content in nuclear DNA found no significant differences between the cerebral cortex and cerebellum of individuals with Down’s syndrome, or Alzheimer’s disease compared to neurotypical controls (Seidl et al. 1997). However, the widely accepted understanding that urinary 8-oxodG is derived from nuclear DNA has been questioned recently (Evans et al. 2016) and may account for this inconsistency. Žitňanová et al. (2004) reported increased uric acid levels in the plasma of individuals with DS versus controls (348.56 ± 22.78 versus 284.00 ± 20.86) and significantly decreased levels of hypoxanthine and xanthine (6.35 ± 0.31 and 1.02 ± 0.23 μmol/L versus 7.83 ± 0.59 and 2.43 ± 0.66 μmol/L) in children with DS versus controls. Additionally, significantly higher levels of allantonin were measured in the plasma of individuals with DS compared to healthy controls (18.58 ± 2.27 and 14.07 ± 1.07 μmol/L, respectively, p = 0.03).

Results from Belardinelli et al. (2008) were consistent with this finding, while also reporting significantly lower lymphocyte and platelet levels of coenzyme Q10 in populations with cardiovascular conditions that are associated with high oxidative stress (for instance, chronic heart failure and indicators of coronary risk). Taken together, these results indicate that oxidative stress may be associated with DS. Interestingly, supplementation with coenzyme Q10 has been shown to decrease biomarkers of oxidative stress (Mousavinejad et al. 2018), although the use of the TBARS assay, and assessment of extracellular GPx and SOD may draw the robustness of these findings into question (see “Discussion”, and Table 1).

#### Intellectual Disability

In a group of adult individuals with intellectual disability, free from conditions and medication regimens, which may increase inflammation, levels of inflammatory molecules and oxidative stress were measured and compared with a control group (Carmeli et al. 2012). Cytokine interleukins 1 and 6 (IL-1α, and IL-6), nitric oxide, and oxidative stress were measured in plasma and capillary blood specimens taken from all participants using enzyme-linked immunosorbent assay (ELISA), reverse transcription and polymerase chain reaction (RT-PCR) and the Western blot. The results indicated that IL-1α and IL-6 were elevated, nitric oxide was upregulated, and an increased level of oxidative stress was found, compared to controls (Carmeli et al. 2012).

## Discussion

The above literature investigating the role of oxidative stress in ASD and intellectual and developmental disability appears to indicate a strong association. However, to date, little attention has been given to the rigor and suitability of the biomarkers and assays that have been utilized in reaching this conclusion. Indeed, there appears to be some confusion between oxidative and nitrosative stress. To help clarify, the species which may contribute to oxidative stress include: the hydroxyl radical (^·^OH—note the superscript ‘dot,’ representing the lone electron accompanies the O of oxygen, reflecting its location in the hydroxyl radical), the reactivity of which is so great that it reacts very close to where it is formed; superoxide (O_2_^·−^) and the non-radical, hydrogen peroxide (H_2_O_2_), both of which are less reactive than ^·^OH. Reactive nitrogen species (RNS) are, as the name suggests, reactive, nitrogen-containing molecules, such as nitric oxide (NO^·^), which is relatively unreactive and peroxynitrite (ONOO– ), which is a powerful oxidant, and widely damaging to many biological molecules (reviewed in Di Meo et al. 2016). Like ROS, RNS have a role in normal cellular function, and pathophysiology and our current understanding has led to the development of the concept of oxidative eustress (beneficial) and oxidative stress (or distress; Sies 2017) and the same appears to apply to nitrosative stress (Moldogazieva et al. 2018).

Biomarker assays must be consistent, reliable, and reproducible to be considered legitimate, and within the oxidative stress field, considerable effort has been made to identify the most appropriate biomarkers, and standardize their measurement (Griffiths et al. 2002). The current literature on ASD, and related studies, displays varying standards of what constitutes a biomarker of oxidative stress, and methodologies used. Herein, we aim to offer some limited guidance in this regard (summarized in Table 1), and direct readers to publications which can further inform on biomarker and assay choice. As it is challenging to assess oxidative stress directly, biomarkers are chosen from the interaction of ROS with cellular biomolecules (e.g. proteins, lipids, nucleic acids), and are invariably a major oxidation product of the biomolecule. These biomarkers should be measured by an assay that is sensitive, reproducible, precise, and robust. To give the assays practical use, in terms of human biomonitoring, they should be applicable to use with easily attainable (ideally non- or minimally invasive), and realistic quantities of tissue, or biofluid.

### Proteins

Various biomarkers of protein modification have been reported in the literature, for example, tyrosine products 3-CT, a specific marker of myeloperoxidase-catalyzed oxidation, and 3-NT, a product of tyrosine nitration mediated by reactive nitrogen species (Ahsan 2013). Amino acids with side chains which are aromatic and or sulfhydryl in nature tend to be the most commonly oxidized but this also places them at risk from artefactual production. Within the ASD literature, HPLC with electrochemical detection (HPLC-EC) has been used to detect 3-CT, and 3-NT (Rose et al. 2012), with immunoslot-blot used for the detection of 3-NT (Ansari and Scheff 2010).

Cysteine and its derivatives, such as S-adenosylhomocysteine, homocysteine, and cystathionine, and methionine (and methionine derivatives, including S-adenosylmethionine) can be measured with HPLC-EC (James et al. 2004). The rationale here appears that as the free thiol group of cysteine readily undergoes reversible oxidation to form a disulfide, loss of cysteine and its derivatives can be viewed as a biomarker of oxidative stress. Of course, a more direct, rather than an inverse biomarker of oxidative stress would be preferred, and the ratio of free, reduced cysteine to oxidized cysteine has also been studied (Rose et al. 2012). Since cysteine is highly susceptible to oxidation, artifact induced during sample workup is a concern. Thiol quantification has limitations, as it simply reflects changes in reduced thiol content, which may not be solely derived from oxidative stress. Lacking the quantification of the specific oxidized product(s) leaves this analysis as suboptimal and should be avoided.

Generally, HPLC-EC is an acceptable method for quantifying protein carbonyls (PCs), although there is a risk of artefact production if acid hydrolysis of the proteins is used (Griffiths et al. 2002). An understanding of the assay and the chemistry of these biomarkers can mitigate this issue. Immunoslot-blot has also been used to quantify PCs, as a marker of global protein oxidation (Ansari and Scheff 2010). This, together with ELISA (Ghezzo et al. 2013), suffers from the weakness which can be inherent with the use of antibodies in quantitative assays e.g. cross-reactivity, lack of a calibration curve, although they may offer a relatively simple means to detect a target molecule in a complex matrix, such as urine, they require only a small volume of sample, and can be high throughput. In general, immunochemical approaches tend to be less favored, often due to cross-reactivity of the primary antibody with non-target compounds, plus they are, at best, semi-quantitative, whereas chromatographic techniques offer absolute quantification. That said, immunochemical techniques can provide localization of the damage within cells or tissues, which chromatographic methods cannot. 2,4-dinitrophenylhydrazine can bind to PCs to form a stable 2 4-dinitrophenyl hydrazone product, which can then be detected by an antibody (Yang et al. 2006) and has been used to study ASD (Forster et al. 1996). Although this approach has been shown to be valid for the study of purified proteins (Cao and Cutler 1995), it may suffer the same weaknesses as other antibody-based methods (see above). PCs have also been determined spectrophotometrically (Yenkoyan et al. 2018; MacFabe et al. 2008) but requires a large volume of sample (approximately 2 mg aliquots are needed for each sample) and gives little information about the specific proteins affected (Castegna et al. 2003). ELISA is sensitive, reproducible, and can quantify specific proteins (Buss et al. 1997; Carty et al. 2000). A more fundamental weakness of the assessment of PCs is that they may be affected by dietary intake (Adachi and Ishii 2000; Carty et al. 2000; Funabiki et al. 1999; Srigiridhar and Nair 2000). Despite these limitations, PCs appear to be the best protein-derived biomarker of oxidative stress.

### Lipids

Lipid peroxidation is a common consequence of oxidative stress, and several assays are available for its assessment. Analysis of MDA, TBARS, lipid peroxides (LPO), isoprostanes, and α, β-unsaturated hydroxyalkenals (specifically 4-hydroxy-2-nonenal, 4-HNE) have been reported in the ASD literature. The TBARS assay has become synonymous with MDA measurement. However, despite the popularity of the TBARS assay, unless it is combined with HPLC–UV, then the assay, as the name suggests, will detect all TBA-reactive substances (e.g., reducing sugars, deoxyribose, methionine, glutamic acid, etc.), not all of which may be derived from lipid peroxidation. Therefore, TBARS is not specific for MDA, although the MDA-TBA conjugate can be identified, and quantified, by HPLC–UV, and is the method of choice when assessing TBARS. Therefore, the simple colorimetric TBARS assay is fundamentally flawed and should be avoided as a means to assess oxidative stress, especially when used as the sole biomarker (Ansari and Scheff 2010; Ghezzo et al. 2013; Yenkoyan et al. 2018; Zoroglu et al. 2004). In terms of artefactual sources of MDA, diet affects MDA determination (Agarwal et al. 1994; Dixon et al. 1998; Mateos et al. 2005), which raises fundamental questions over its usefulness.

4-HNE is a major product of lipid peroxidation, but it is quickly removed from cells as several enzymes control its metabolism (Esterbauer et al. 1991). Use of both immunoslot-blot (Ansari and Scheff 2010) and Western blotting (Pecorelli et al. 2013; Zhang et al. 2008) have been reported in the ASD literature for the assessment of 4-HNE but, as they both rely upon anti-4-HNE antibodies, they are likely to be prone to the same short-comings as noted above. HPLC with either -EC, chemiluminescence, or mass spectrometric detection are the best options. GC–MS is also acceptable. These methods are highly sensitive and may provide absolute quantification, but possess the risk of artefactual production of 4-HNE during sample workup.

Isoprostanes form when free radicals interact with arachidonic acid, primarily (Milne et al. 2005). In the ASD literature, total isoprostanes have been measured via gas chromatography, with negative ion chemical ionization tandem mass spectrometry technique (Leoncini et al. 2011), ELISA (Ghezzo et al. 2013), and a third study that did not disclose their methodology (Grillo et al. 2013). Isoprostanes in plasma are excreted rapidly and have a short half-life, making their measurement more challenging (Basu 2007; Cracowski 2006). However, this can be overcome by using urine samples, with a caveat that localized peroxidation in the kidney may occur. Morrow and colleagues showed that this artefactual peroxidation can be excluded from the quantification by measuring a metabolite of the isoprostane in question instead (2,3-dinor-5,6-dihydro-8-iso-PGF_2α_; Morrow et al. 1999), which was not done in the study cited above. GC–MS and LC–MS/MS are considered the assays of choice for this biomarker, with LC–MS/MS the gold standard. As with seemingly all biomarkers of lipid peroxidation, it appears that diet can affect levels (Astley et al. 1999; Chopra et al. 2000; Gopaul et al. 2000; Porkkala-Sarataho et al. 2000; Rust et al. 1998; Thompson et al. 1999; Upritchard et al. 2000; Wander and Du 2000; Watanabe et al. 1979; Young et al. 1999).

### Nucleic Acids

The oxidation product of 2′-deoxyguanosine, 8-oxodG is a popular biomarker of oxidative stress which is widely measured, mostly in DNA, and urine (Griffiths et al. 2002). Amongst the ASD literature 8-oxodG has been measured by HPLC with UV (Liang and Pate 2004), EC (Seidl et al. 1997), and MS (Rose et al. 2012) detection, and also ELISA (Ghezzo et al. 2013). Perhaps the greatest issue affecting the measurement of oxidatively modified DNA is the possible generation of artefact during sample workup (e.g. DNA extraction and hydrolysis) and analysis (e.g. during derivatization for GC–MS; Halliwell, 2000), which is understood to be responsible for the discrepancies in the levels of 8-oxodG over a range of two orders of magnitude (Collins et al. 1997). There have been many concerted efforts to minimize artefact, perhaps most notably from the European Standards Committee on Oxidative DNA Damage (ESCODD; Lunec 1998). The work of ESCODD, and others, led to the development of protocols which minimized artefactual oxidation (Ravanat et al. 2002), and gold-standard procedures, such as LC–MS/MS being recommended (Møller et al. 2012). Again, the ELISA approaches for 8-oxodG may suffer from the same issues as the immunochemical methods described above, although some improvements have been made, at least for urinary 8-oxodG (Rossner et al. 2013, 2016), these have not been used in the studies reported in the ASD literature. The work of ESCODD, in part, increased the popularity of single-cell gel electrophoresis (comet assay), as a valuable means to assess oxidative stress with minimal risk of artefact, and led to the formation of the European Comet Assay Validation Group (Ersson et al. 2013; Forchhammer et al. 2012; Godschalk et al. 2014) to support assay validation. However, to date use of the comet assay has not been reported in the ASD literature, despite it being a simple, rapid, and sensitive method for detecting DNA strand breaks, and oxidatively generated modifications to DNA nucleobases.

Measurement of 8-oxodG in urine possesses a number of advantages over cellular DNA, it is entirely non-invasive, and with little risk of an artefact, or underestimation, if key workup steps are adhered to (Shih et al. 2018). The work of the European Standards Committee on Urinary (DNA) Lesion Analysis (ESCULA), and others, ruled out dietary influence on urinary 8-oxodG levels (Cooke et al. 2005; Gackowski et al. 2001), identified LC–MS/MS to be the gold standard technique (Barregard et al. 2013; European Standards Committee on Urinary (DNA) Lesion Analysis et al. 2010), and showed that spot urine samples, with creatinine correction, can substitute for the more challenging 24 h collections (Barregard et al. 2013). Increasingly it appears that the oxidatively modified RNA product, 8-oxo-7,8-dihydroguanosine might be a more clinically relevant biomarker of oxidative stress (Broedbaek et al. 2013; Kjær et al. 2017), compared to 8-oxodG, or indeed MDA (Shih et al. 2018) but, again, this has yet to translate into the ASD literature.

### Antioxidants

In the ASD literature, superoxide, GSH/GSSG, GSH alone, glutathione reductase, and total antioxidant capacity (TAC) have all been measured individually and interpreted as being reflective of total oxidant/antioxidant levels. The rationale for this presumably being that whatever affects the level of one antioxidant, will reflect them all, and hence any one of them may be measured, and reflect the total cellular antioxidant capacity. The basis of this presumption does not appear to be present in the literature, in fact, the opposite seems to be indicated (Griffiths et al. 2002).

Superoxide has been measured in postmortem brain tissue by electron paramagnetic resonance spectroscopy (Ho et al. 2015). The Mn^2+^-diaminobenzidine method may be used, for the detection of superoxide in stimulated cells, or perfused tissues (Babbs 1994; Steinbeck et al. 1993), but seemingly not for in vivo human studies. Measurement of total levels of a specific antioxidant (e.g. GSH) would seem to provide limited information on levels of oxidative stress, not least due to the multiplicity of other compounds that may act as antioxidants, or free radical scavengers. Indeed, it may be argued that any compound is a free radical scavenger if present in sufficient quantity. Comparing the GSH/GSSG ratio might be a little more informative but is impacted by diet and metabolism (Rojas et al. 1996). Thus far, there is insufficient knowledge about both the active and oxidized form to be trusted fully (Griffiths et al. 2002). GSH/GSSG levels have been measured by HPLC with UV (James et al. 2004), or by HPLC with EC (Rose et al. 2012, 2014) or fluorometric detection (Ansari and Scheff 2010). However, HPLC–UV has been shown to detect other substances that are derivatized, and co-elute, along with GSSG, leading to a lack of specificity and over-estimation of values (Sian et al. 1997). A logistical consideration is that GSH levels decrease within minutes of sample collection, up to 70% decrease within 15 min (Sian et al. 1997), and so the samples must be processed immediately upon collection.

Perhaps more reflective of cellular antioxidant status is TAC, comprising either ferric reducing antioxidant power, oxygen radical absorbance capacity, and Trolox-equivalent antioxidant capacity. In the ASD field, these have been applied to plasma and serum (Campos et al. 2010), though concerns have been raised related to estimating this parameter. The main concern with TAC is that some antioxidants, once oxidized, are very easily reversible. Since these reactions are easily reversible, the indicator can be oxidized; therefore, the results of the reaction actually demonstrate the capacity of the antioxidant to react with the oxidized form of the indicator, rather than with the oxidant itself (Bartosz and Bartosz 1999; Griffiths et al. 2002). Also, whether the TAC is intracellular (e.g. plasma or serum) or extracellular is expected to be important—intracellular levels of antioxidants are likely to be more influential on intracellular redox status, and therefore cell function. The next question is does TAC truly assess total antioxidant capacity? After all, the contributions from enzymic antioxidants (e.g. superoxide dismutase and catalase), and lipophilic antioxidants are not assessed, and they are disproportionally influenced by uric acid levels, all of which are major limitations (Ferrari and Ferrari 2011). Furthermore, a review of the concept of TAC, concludes that, given that the major antioxidant defense is enzymatic, the use of the term “total” is not completely accurate (Sies 2007). One proposed, alternative approach to assessing TAC is to measure the cellular response to a standardized oxidant challenge. For example, we have reported the measurement of DNA damage in cells before and after exposure to 50 µM hydrogen peroxide, demonstrating profoundly attenuated antioxidant defenses in cells with a mutated *SECISBP2* gene (Schoenmakers et al. 2010). This approach takes into account the majority enzymic antioxidant defenses, demonstrated by their attenuation through the loss of selenium from various proteins with antioxidant properties e.g. the glutathione peroxidases and thioredoxin reductases.

### ROS Dsetection

As noted elsewhere in this review, the reactivity of ROS and RNS precludes their direct detection, but it is possible to indirectly assess their production using ROS/RNS-sensitive agents which invariably become fluorescent upon interaction with ROS/RNS. Such assays are invariably limited to in vitro, cell culture studies, so not applicable to many of the studies reported in this review but, for completeness, it is worth noting that the limitations of these assays, which have been well described elsewhere (Kalyanaraman et al. 2012, 2016), and include a lack of specificity for particular ROS/RNS, confusion over specificity by authors, and manufacturers, and contributions from non-ROS/RNS sources, such as reactive sulfur species (De Leon et al. 2007; Olson et al. 2018).

Given the current state of literature, it is evident that although the pathogenesis of ASD and IDD is yet to be identified, oxidative stress has been clearly implicated in their etiology. However, questions exist as to what constitutes a biomarker of oxidative stress, and the methodologies for its assessment. In light of this, it is necessary to consider how future studies can be designed to strengthen the field.

Moving forward, studies on oxidative stress in ASD and IDD should be appropriately powered but, perhaps more importantly, selection of the chosen biomarkers should be based upon careful review of the literature rather than, for example, availability of a commercially available kit. These biomarkers must have high specificity, stability, and have been validated as being accurate proxies for oxidative stress, free from unrelated influence such as contribution from the diet. The assay should be robust, and validated, with published limits of detection, and reproducibility in the biological matrix to be studied. Assay-specific best practices should be adopted e.g. the use of quantifier and qualifier ions for each analyte, in the case of mass spectrometry. The matrix in which the biomarker is to be studied should also be considered, for example, are intercellular antioxidant levels more relevant than extracellular? What variables may affect matrix biomarker levels—in the case of urine, are spot or 24 h collections most accurate, and how can variation in urine concentration be normalized?

Sample collection, processing, and storage are also possible sources of error. Institutional review boards are likely to favor non- or minimally-invasive sample collection methods, such as urine, or finger-pricks of blood, but these matrices may not work for all biomarkers. Sub-optimal methods for the extraction of DNA may lead to artefactual oxidation (Hamilton et al. 2001), as might long term storage of large (e.g. 5 mL) volumes of whole blood (Al-Salmani et al. 2011).

In conclusion, there appears to be a high number of studies purporting to measure biomarkers of oxidative stress in ASD/IDD, which have used sub-optimal methodology, choice of biomarkers, and/or sample collection/processing. Therefore, whilst the weight of evidence supports a role for oxidative stress in ASD/IDD, the number of well designed, executed studies that support this evidence is relatively small in comparison, making the overall literature less than robust. Only with the issues highlighted in this review considered, will the data from well-phenotyped individuals lead to robust conclusions as to the role of oxidative stress in ASD and other neurodevelopmental disorders.
